# Humoral and cellular immune responses in cattle upon *Clostridium chauvoei* vaccination and challenge

**DOI:** 10.3389/fimmu.2025.1584168

**Published:** 2025-05-20

**Authors:** Andrea Rossi, Julio Guarnaschelli, Analía Rial, María Moreno, Mariana Rivera-Patron, Andrés Iriarte, José A. Chabalgoity

**Affiliations:** Unidad Académica de Desarrollo Biotecnológico, Instituto de Higiene, Facultad de Medicina, Universidad de la República, Montevideo, Uruguay

**Keywords:** *C. chauvoei*, cattle, polyclostridial vaccine, humoral immunity, cellular immunity, IFN-γ, vaccine efficacy

## Abstract

**Introduction:**

*Clostridium chauvoei* is the causative agent of blackleg, a severe disease in cattle. Vaccination reduces disease incidence but the immune mechanisms that underlie vaccine-induced protection remain poorly understood, particularly the role of cellular immunity. In this study we characterized the humoral and cellular immune responses induced by a polyclostridial vaccine and assessed their correlation with protection against a *C. chauvoei* challenge.

**Methods:**

Eleven six month old Hereford calves, seronegative for anti *C. chauvoei* antibodies, were randomized into vaccinated (n=8) and control (n=3) groups. Vaccinated animals received two doses of the vaccine at days 0 and 42-days. All animals were intramuscularly challenged with *C. chauvoei* spores (8,000 LD50) at day 69 post vaccination and monitored for clinical outcomes. Blood samples were collected at pre-vaccination and pre- and post-challenge. Humoral responses were quantified by specific in-house developed ELISA. Cytokine gene expression was measured in whole-blood (RT-qPCR for IFN γ, TNF α, TGF β1, IL 4, IL 17A, IL 12B, IL 10) upon antigenic stimulation.

**Results:**

While vaccination protected cattle upon challenge and all animals survived, unvaccinated controls developed severe disease and died. Vaccination induced a strong specific antibody response although with inter-individual variation as well as a specific cytokine profile characterized by increased expression of IFN-γ, TGF-β1, and IL-4. Post-challenge, IFN-γ and IL-12B expression declined in vaccinated animals, but TGF-β1 persisted. High pre-challenge IgG, IFN-γ, and TGF-β1 were associated with protection, whereas increased IL-12B post-challenge was associated with disease severity.

**Discussion:**

These findings demonstrate a coordinated interplay between humoral and cellular immune responses in vaccine-induced protection, with IFN-γ emerging as a potential biomarker in conjunction with antibody titre. The study provides a deeper further understanding on the immune mechanisms underlying vaccine-mediated protection against *C. chauvoei* and shall be relevant for the development of more effective vaccines.

## 1 Introduction

*Clostridium chauvoei* is a Gram-positive bacterium, strictly anaerobic, spore-forming that is spread worldwide. *C. chauvoei* is the etiological agent of blackleg, a severe infectious disease predominately affecting cattle, characterized by rapid and severe myonecrosis, systemic toxemia, and high mortality. Infection occurs via spore ingestion or wound entry that persistently contaminate pasture soils. Spores germinate under anaerobic conditions (e.g., muscle trauma), producing vegetative cells that release toxins (e.g., sialidase, flagellin), causing blackleg. Sporulation within necrotic tissues facilitates environmental spread, perpetuating transmission. Blackleg primarily affects cattle from 6 months to 2 years of age and in good nutritional and physical condition, and causes significant lost in livestock production due to its high lethal rate (1, 2). Despite being one of the oldest recognizable diseases in cattle, the exact mechanisms of blackleg’s pathogenesis remain largely unknown (1, 3, 4).

Vaccination is the main strategy for preventing blackleg and other clostridial disease. Polyclostridial vaccines have been widely used since 1930 and have significantly reduced the incidence of the disease in vaccinated cattle in areas where it is regularly used (2, 5). In general, commercial vaccine manufacturers recommend a similar vaccination schedule consisting of a priming dose, a booster at 4 weeks, and annual revaccination. However, the timing may vary depending on the production system and physiological stage. In beef calves, primary immunization typically begins at 3 months of age, whereas in dairy systems, it usually occurs between 2 and 3 months of age. Annual revaccination in adult cattle is often aligned with reproductive cycles or calving. In endemic regions or high-risk settings, administering the vaccine 4 to 8 weeks before calving can enhance passive immunity in newborn calves. Vaccine-induced protection against *C. chauvoei* has been associated with the induction of neutralizing antibody responses (2, 6–8), and regulatory agencies have established minimum antibody levels to ensure the protection of target species (9, 10). In this sense, it is known that commercial vaccines induce a systemic response with high levels of specific IgG antibodies; however, previous studies have reported the induction a heterogenous humoral response with antibody levels significantly varying between animals (stratified as high and low responders) and a shorter duration than expected. Antibody levels decline significantly, becoming undetectable for most vaccine antigens at 6 months post-vaccination (11–15). Scientific evidence demonstrating the efficacy of polyclostridial vaccines to protect cattle against *C. chauvoei* is extremely scarce (5). However, the absence of major outbreaks suggests that long-term memory responses ensure protection. In this regard, our group found that annual revaccination induces a rapid and strong increase in anti-*C. chauvoei* levels in cattle (12). Altogether, these data suggest that cellular immune responses might play an important role in protection, making it essential to advance in current knowledge of the immunological mechanisms underlying vaccine-induced protection against *C. chauvoei* and other clostridia.

Historically, pathogenic clostridia were considered to be exclusively extracellular pathogens, but recent studies have shown that *C. chauvoei* can survive within bovine monocyte-derived macrophages. Infected macrophages display different responses: vegetative forms of *C. chauvoei* induce pro-inflammatory cytokines IL-12 and IL-23, while spores trigger an anti-inflammatory response with secretion of IL-10 and TGF-β (16). Similar to *C. difficile* and *C. perfringens*, *C. chauvoei* spores appear to evade detection due to their low metabolic activity, allowing them to persist and spread without eliciting an inflammatory response (17, 18). In contrast, a recent study showed that vegetative *C. chauvoei*-infected macrophages produce apoptotic bodies and express iNOS and TNF-α, indicating that inflammation-induced apoptosis may help the bacteria evade immune responses by depleting phagocytic cells at the site of infection (19). A pro-inflammatory response with high levels of IFN-γ expression has also been reported in *C. chauvoei*-stimulated PBMCs from vaccinated sheep (20). On the other hand, clostridia such as *C. difficile* and *C. perfringens* induce an inflammatory response with high levels of IFN-γ expression (21–25).

Previously, we formulated an experimental polyclostridial vaccine with a composition identical to a commercially available one (Prondivax Multi C10, Prondil S.A.) considered as a reference vaccine and demonstrated that the experimental vaccine induced a humoral response to each of the clostridium antigens similar to that of the market-approved reference vaccine (11). Based on these findings, we aimed to characterize both humoral and cellular immune responses induced by this experimental polyclostridial vaccine in cattle, and to assess the immunological changes after a lethal challenge with *C. chauvoei* spores. We evaluated specific-antibody responses, cytokine gene expression profiles, and lymphocyte proliferation patterns pre- and post-challenge. Our findings provide a deeper understanding of the immune mechanisms underlying vaccine-induced protection, thus contributing to the development of more effective vaccination strategies against *C. chauvoei*.

## 2 Materials and methods

### 2.1 Animals

Eleven 6-month-old Hereford bovines of both sexes, weighing around 150 kg, were used for the study. All animals were purchased from a local provider and had not received previous Clostridial vaccine. The cattle were selected considering the absence of detectable levels of anti-*C. chauvoei* antibodies before the beginning of the experiment. Before the study, cattle were treated with an anti-parasitic agent (Ivomec^®^, 1cc per 33 kg of weight).

### 2.2 Vaccine and antigen

The polyclostridial vaccine used in this trial (from now on “the experimental vaccine”) was previously described by us (11). The 9-valent vaccine contains toxoids of *C. novyi* type B; *C. perfringens* types A, C, and D; *C. septicum*, *C. sordellii*, and *C. tetani*; and a bacterin from *C. chauvoei*, and the adjuvant aluminum hydroxide.

The C*. chauvoei* antigen used to perform specific ELISA and *in vitro* cell stimulation was prepared from the strain used to formulate the vaccine. The *C. chauvoei* strain was grown as detailed in Rossi et al. (2018) (11) and the antigen was prepared according to Santos et al. (2021) (20) with modifications. Bacteria were collected by centrifugation at 9,000 x g for 15 min at 4°C and the pellet was washed twice with Phosphate Buffered saline (PBS). Finally, the pellet was resuspended in PBS, sonicated 10 times (at amplitude 60 hz, 20 seg on and 20 seg off) on ice, until the sample was clear, and centrifugated at 12,000 rpm for 10 min at 4°C. The supernatant was quantified and stored at -20°C until use (20).

### 2.3 Vaccination and challenge assay

The bovines were randomly divided into two groups: eight animals received 2 doses (4 mL/dose) of the vaccine at 0 and 42 days (vaccinated group), and three animals received PBS (control group).

At 69 days post-vaccination (dpv), 27 days after the second dose of polyclostridial vaccination, all animals were intramuscularly challenged with 8,000 LD_50_ (50% lethal dose for mice) of a suspension of *C. chauvoei* spores in the hind limb. To standardize the clinical evaluation of the animals, a scoring system was developed to quantify disease severity based on general health status (including lethargy, depression, loss of appetite, muscle tremors, and generalized weakness), rectal temperature, the presence and severity of local inflammatory lesions (heat, pain, swelling, and edema), and functional impairment of the inoculated limb. The scale was defined as follows: 0 = no clinical signs (asymptomatic); 1 = very mild signs; 2 = mild signs; 3 = moderate signs; 4 = severe signs; and 5 = terminal stage of disease associated with *C. chauvoei* infection. The normal rectal temperature range in calves is 39.0–39.5°C. Therefore, a temperature above 40.5°C was considered indicative of fever (26). All animals were monitored daily for 72 hours after challenge. Vaccinated cattle were kept in pens for 15 days to confirm the absence of symptoms.

Animals were bled before receiving the first vaccine dose at 0 dpv (Pre-Vaccinated time), before the challenge at 69 dpv (Pre-Challenge time), and 24 hours after the challenge at 70 dpv (Post-Challenge time). Whole blood was collected into lithium heparin tubes (BD) to study the cellular response, and into dry tubes to analyze the humoral response. Blood samples were kept at 4°C and processed within 4 hours of extraction.

All animals were housed at a dedicated experimental facility located in the Department of Lavalleja, Uruguay, which operates in compliance with both national and institutional regulations for animal care and use. Throughout the study period, animals were maintained in corral with unrestricted access to food and water. Vaccination and challenge procedures were conducted on-site under continuous veterinary supervision. All the experimental procedures complied with current national and institutional animal welfare regulations and were approved by the Honorary Commission for Animal Experimentation (CHEA) of Uruguay, under approval identification CEUA-FMED n°1522.

### 2.4 Antibody-level determination assays

Levels of anti-*C. chauvoei* antibodies were evaluated using an in-house specific ELISA. Briefly, high binding ELISA microplates (Greiner Bio-One) were coated with 100 μL/well of *C. chauvoei* antigen (40 μg/mL final concentration) in acetate buffer (pH 4.5) and incubated for 2 hours at 37°C. Plates were then washed with PBS-T (0.1% Tween 20 in PBS). A standard curve was prepared in 0.5% non-fat dry milk (ndM) in 0.2% Tween 20 PBS (0.5% ndM-PBS-T) using a pool of *C. chauvoei*-positive sera from bovines vaccinated with a polyclostrial vaccine that included *C. chauvoei* from a previous study (11). A standard curve was included in each plate in a two-fold dilution series. Test sera were evaluated in six 4-fold dilution series, starting at 1/100 in 0.5% ndM-PBS-T. After washing, the standard curve and test sera (100 μL/well) were added in duplicates and incubated overnight at 4°C. Following washing, plates were incubated with 100 μL of a 1-in-5,000 dilution, in 0.5% ndM-PBS-T, of rabbit anti-bovine IgG (whole molecule)-peroxidase antibody (Sigma) at 37°C for 1 h. Next, 100 μL/well of the peroxidase substrate Fast OPD (Sigma) was incubated in the dark for 10 min. The reaction was stopped with 30 μL/well of 3M sulfuric acid. Optical density (OD) values were measured at 492 nm in an ELISA microplate reader (Dynex Technology).

Antibody titers were expressed as arbitrary units (AU) as described before (11). A standard curve was generated using a pooled bovine serum positive for anti-*C. chauvoei* antibodies. This pooled serum was prepared by mixing equal volumes of sera from 10 animals immunized with a commercial polyclostridial vaccine, and was designated a reference value of 100 AU/mL. The standard curve was constructed through ten serial dilutions, initiated at a 1:100 dilution followed by nine sequential two-fold serial dilutions. To assign an AU value to each test sample, three dilutions of each test serum (with OD values within the linearity range of the standard curve) were chosen and interpolated into the standard curve. These values were averaged, and SD and CV were calculated, in this case, the intra-assay CV was less than 15%.

The *cut-off* value was calculated as the average specific antibody titer of 50 naïve cattle plus 3 standard deviations (SD). All test samples above *cut-off* value were considered positive in response to vaccination.

### 2.5 Whole blood *in vitro* stimulation

Aliquots of the heparinized blood (1 mL) from all animals, pre- and post-challenge, were dispensed into individual wells of 48-well tissue culture plates (Greiner Bio-One) containing *C. chauvoei* antigen (15 μg/mL) or PBS without Ca^2+^Mg^2+^ (Sigma Aldrich). 10 μg/mL of Concanavalin A (Con A, Sigma Aldrich), was used as a positive control for each blood sample. Cultures were incubated for 6 h at 37°C in a humidified atmosphere with 5% CO_2_.

After the stimulation, red blood cells were removed by using EL buffer (QIAGEN) in a 1:5 ratio (v/v). Samples were mixed by inversion, incubated on ice for 10 min, and centrifuged at 500g for 10 min at 4°C. Pellets were washed twice with PBS without Ca^2+^Mg^2+^ (Sigma Aldrich). Peripheral blood leukocytes (PBLs) were homogenized in 1ml of TRIzol^®^ Reagent (Invitrogen) and kept at -80°C until RNA extraction.

### 2.6 Gene expression analysis

Total RNA from pre- and post-challenge PBLs was extracted by using TRIzol reagent (Invitrogen) according to the manufacturer’s instruction. RNA concentration was measured using a NanoDrop ND-2000 spectrophotometer (NanoDrop Technologies, Wilmington, DE, USA). Only RNA samples with an A_280_/A_260_ ratio in the range of 1.8-2.0 and an A_260_/A_230_ ratio in the range of 2.0-2.2 were used for cDNA synthesis. Total RNA (1 μg) was treated with 0.4 U DNase I (Invitrogen) to remove residual DNA, and then reverse transcribed using Moloney murine leukemia virus (M-MLV) reverse transcriptase (Invitrogen), random primers (Invitrogen) and RNaseOUT (Invitrogen) in a final 20-μL reaction mixture.

Following retrotranscription, quantitative PCR (qPCR) for IFN-γ, IL-17A, IL-10, IL-1α, IL-4, TNF-α and TGF-β1 mRNA was conducted using QuantiTect^®^ SYBR^®^ Green PCR Kit (Qiagen) in an ABI 7900 HT (Applied Biosystems) thermocycler. β2-Microglobulin (B2M) encoding gene was used as the housekeeping gene. Primer sequences used are shown in **Table 1**. Cycle program was as follows: initial incubation of 15 min at 95°C; followed by 40 cycles of 15 s at 95°C, 1 min at 60°C with data acquisition; and finally, a melt curve with a ramp from 60 to 95°C at 1°C/s. Melt curve analysis was performed to identify and exclude reactions with alternative amplicons.

**Table 1:** Primer pairs used for qPCR.

Target Gene	Primers (5´→3´)		Amplicon size (bp)
	Forward	Reverse	
β2M^*^ (27)	agacacccaccagaagatgg	tccccattcttcagcaaatc	98
IFN-γ (27)	gcgcaaagccataaatgaac	cttctcttccgctttctgag	78
IL-17 (28)	tccatctcacagcgagcacaag	agccaccagactcagaagcagtag	113
IL-4 (29)	gccacacgtgcttgaacaaa	tgcttgccaagctgttgaga	63
TNF-α (29)	tctaccagggaggagtcttcca	gtccggcaggttgatctca	68
TGFβ1 (30)	cgagccctggacaccaacta	aggcagaaattggcgtggta	137
IL-12B	catcagggacatcatcaaacca	ccacctgccgagaattctttaa	74
IL-10	accagcctgccccacat	ggtcaacagtaagctgtgcagttg	99
IL-1α	cacctctctctcaatcagaagtcctt	aggtatccagggacataaacttattca	91

Genes, primer pairs, and product size (bp = base pairs) used for qPCR analysis.

*Endogenous control gene.

Each primer pair was validated, and its efficiency was calculated using a standard curve of six threefold serial dilutions of a representative sample of pooled cDNA (data not shown). Besides, B2M gene was confirmed as a suitable normalizing gene. For this, B2M Ct values of vaccinated and unvaccinated cattle were compared and no significant differences were observed between both groups (*p=0.0707*, Mann-Whitney test). Furthermore, B2M Ct values from *C. chauvoei* antigen-stimulated blood samples were compared against the same unstimulated samples, and no statistical differences were found between both sample types (*p=0.4994*, Mann-Whitney test) (data not shown).

The relative mRNA amount in each sample was calculated using the 2^−ΔΔCt^ method as previously described Livak and Schmittgen (31) where ΔCt = Ct_gene of interest_ - Ct_B2M_. For each cytokine, mRNA level was expressed relative to the average of the unstimulated condition (PBS) of all cattle (vaccinated and unvaccinated) at 0 dpv (calibration condition).

### 2.7 PBMC isolation

Heparinized blood was used to isolate PBMCs with Lymphocyte Separation Medium (LSM) (Capricorn, Inc.), according to the manufacturer’s instructions. Briefly, blood samples were diluted 1:1 with PBS without Ca^2+^Mg^2+^, overlaid onto LSM at a 2:1 ratio, and centrifuged at 1,000 g for 30 min at room temperature with the break-off. The PBMC interface was carefully removed by pipetting, washed twice with 5 mL of PBS without Ca^2+^Mg^2+^, and centrifuged at 400g for 10 min. PBMC pellets were resuspended in RPMI 1640 medium (Capricorn, Inc) supplemented with 10% Fetal Bovine Serum (FBS) (Capricorn, Inc), 2 mM L-glutamine, 50 μM 2-mercaptoethanol (Sigma-Aldrich), and 100 μg/mL penicillin–streptomycin (Sigma-Aldrich). Total viable cells were counted on a hematocytometer, with trypan blue as viability exclusion dye.

### 2.8 Flow cytometry analyses

Cells were immunostained at 4°C in the dark for 30 minutes with antibodies against: CD4-PE (clone CC8), CD8-Alexa 647 (clone CC63) and CD22-biot (clone Mc64-12) followed by a second staining with Strep-PE/Cy7 (all from Bio-Rad Laboratories, Inc.). Optimal antibody concentrations were previously defined by titration. Flow cytometry data were collected on a FACS Canto II cytometer equipped with two lasers (488 and 633nm) and analyzed using FACSDiva Version 6.1.3 software (BD Biosciences, Oxford, UK).

Gate strategy was performed as: Cells (P1 population) were gated in an FSC *vs* SSC dot plot, then, singlets were gated in the FSC-H vs FSC-A dot plot (P2), and finally, CD4^+^ T lymphocytes (CD4-PE), CD8^+^ T lymphocytes (CD8-Alexa 647) and B lymphocytes (CD22-biot_PECy7) populations were defined on specific cell-surface markers expression within P2 (**Supplementary Figure 1**).

### 2.9 Proliferation assay

PBMC were washed three times with RPMI 1640 medium (Capricorn) supplemented with 10% FBS (Capricorn, Inc), 2 mM L-glutamine, 50 μM 2-mercaptoethanol (Sigma-Aldrich), and 100 μg/mL penicillin–streptomycin (Sigma-Aldrich), counted and resuspended at a concentration of 1×10^6^ cells/mL. Cells were labeled with 3 μM CFSE (Sigma-Aldrich) and then seeded in duplicates into 48-well plates (Nunc, IL, USA) at 4×10^5^ cells per well in 500 μL of supplemented RPMI. Cells were incubated for 5 days with Con A (Sigma-Aldrich) or left without stimuli. Cells were collected and stained as described above to assess lymphocyte proliferation by flow cytometry. Cytometry analysis was performed gating on total cells.

Gating strategy to define lymphocyte populations was performed as described above. Cell generation gates were finally defined within each cell population. The number of proliferative cells (events) in each gate was recorded, and the number of parental cells from which each generation of proliferative events derived was calculated.

The proliferation index (PI) was then calculated as: ∑ of proliferative cells/∑ of parental cells and normalized by the non-stimulated condition.

### 2.10 Statistical analyses

The analysis of the immunological parameters evaluated at each time-point (pre-vaccination, pre- and post-challenge) within each group (vaccinated or control) was performed using non-parametric and paired tests (Wilcoxon or Friedman test followed by Dunn test). The comparison between groups (vaccinated *vs* control or protected vaccinated *vs* non-protected unvaccinated cattle) was analyzed using the non-parametric Mann-Whitney test.

To identify high and low responders of the humoral immune response, a three-step analysis was performed. First, bimodality was assessed using Silverman’s test (multimode package, ACR method, R v4.3.1) and Hartigan’s dip test (diptest package, R v4.3.1), both of which rejected unimodality (*p* < 0.05). Second, based on the evidence of bimodality, the K-means clustering algorithm (R v4.3.1) was applied with k=2, following Z-score standardization to ensure data comparability. Finally, the Mann-Whitney test (GraphPad) was used to confirm that the two clusters represented statistically distinct subgroups. A significance threshold of α = 0.05 was applied in all analyses.

To determine associations between immunological parameters at pre- and post-challenge times a Wilcoxon test was performed. Concordance between immunological parameters was calculated using non-parametric Spearman correlation coefficient r.

To compare the PI of the different populations, a pre- and post-challenge paired Wilcoxon test was performed between the animals in the vaccinated group and between the control bovines. Values of *p* < 0.05 were considered statistically significant. All statistical analyses were performed using Prism software (GraphPad, La Jolla, CA, v9.0.2).

## 3 Results

### 3.1 Polyclostridial vaccination protects against *C. chauvoei* challenge

Eight cows were immunized at day 0 and received a booster 42 days post-vaccination (dpv). Three animals received PBS as control. All animals were challenged with spores at 69 dpv. Clinical symptoms and outcomes after a *C. chauvoei* challenge of vaccinated and unvaccinated cattle are shown in **Table 2**. Unvaccinated cows showed severe symptoms (clinical score ›4) associated with *C. chauvoei* infection and all died between 48- and 72 hours post-challenge. Instead, all vaccinated cattle survived the challenge and only showed mild symptoms (clinical score ‹3 at 24- and 48-hours post-challenge), followed by a rapid clinical recovery (**Table 2**).

**Table 2:** Clinical outcomes at 24- or 48-hours post-*C. chauvoei* challenge of vaccinated and unvaccinated cattle.

Group	ID Animal	Condition	Clinical Symptoms (24 h)		Score 24 h	Clinical Symptoms (48 h)		Score 48 h
			Temp (°C)	Observation of lesions		Temp (°C)	Observation of lesions	
Vaccinated	1	Vaccinated	39.9	+	1	40.5	+	2
	2	Vaccinated	39.1	+	1	39.8	+	1
	3	Vaccinated	39.8	-	1	39.2	-	0
	4	Vaccinated	39.3	+	1	39.8	++	2
	5	Vaccinated	39.6	-	1	39.8	-	0
	6	Vaccinated	39.9	+	1	39.2	+	1
	7	Vaccinated	39.8	+	1	39.7	+	1
	8	Vaccinated	39.0	-	0	39.6	-	0
Control	1	Unvaccinated	39.6	+++	4	ND	++++	5
	2	Unvaccinated	40.1	+++	4	ND	++++	5
	3	Unvaccinated	39.3	+++	4	ND	++++	5

Clinical score was created considering the rectal temperature, the animal’s general condition, the presence and severity of local inflammatory lesions (heat, pain, swelling, and edema), and functional impairment of the inoculated limb. The scale was defined as follows: (0): no clinical signs (asymptomatic); (1): very mild signs; (2): mild signs; (3): moderate signs; (4): severe signs; and (5): terminal stage of disease. Lesions were defined as: (-) no visible lesions, (+) mild swelling; (++) moderate swelling, moderate deformation of the limb, moderate edema; (+++) severe swelling, severe deformation of the limb, heat, severe edema; (++++): Loss of function of the inoculated limb. Temp, temperature; ND, not determined.

### 3.2 Antibody response against *C. chauvoei* shows inter-individual variation and is not affected by the challenge

**Figure 1:**
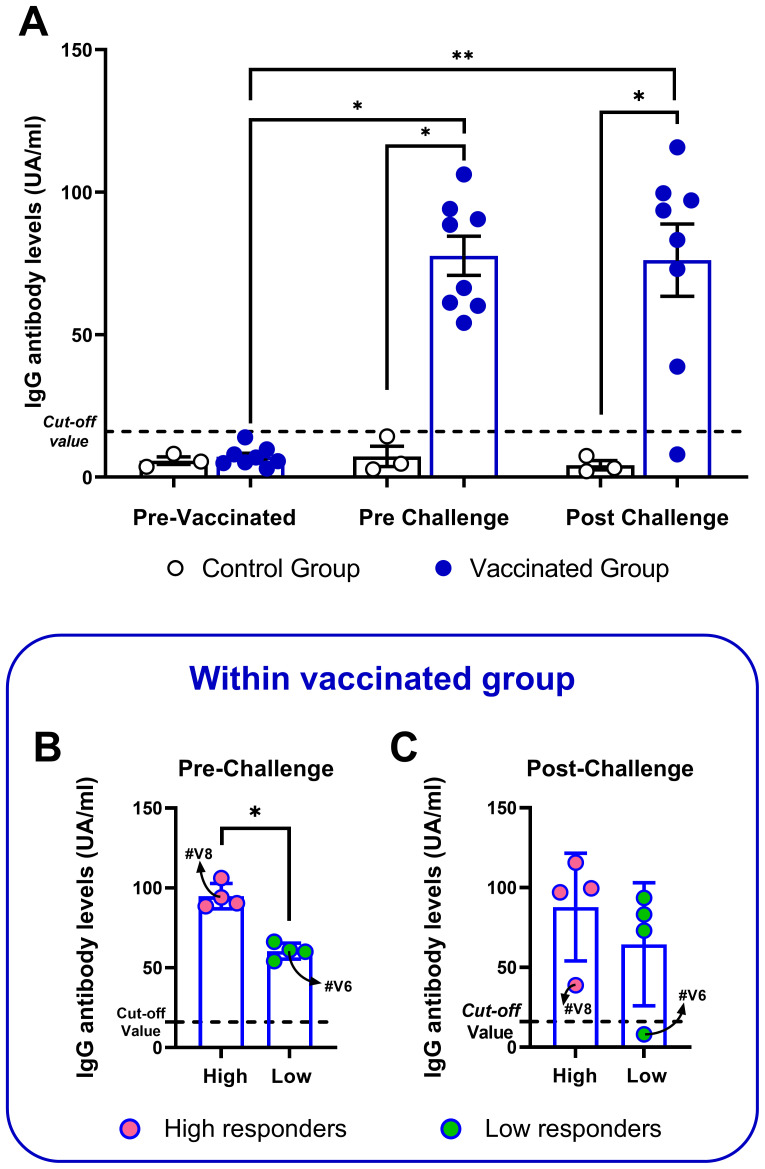
Anti-C. chauvoei IgG levels of vaccinated and control cattle at pre-vaccination and pre- and post-challenge. Bars indicate mean ± SEM and the dots represent individual animals. **(A)** Antibody levels of vaccinated (n = 8) or control (n = 3) cattle at all timepoints. Stratification of vaccinated animals into high and low responders based on their antibody levels pre- **(B)** and post-challenge **(C)**. Temporal comparisons within each group were assessed using nonparametric paired tests Friedman test, followed by Dunn’s *post hoc* test. Intergroup comparisons (vaccinated vs. unvaccinated) were performed using the Mann-Whitney test. (*p < 0.05; **p < 0.01).

The systemic antibody response against *C. chauvoei* was assessed in sera at three different time points, before vaccination (“pre-vaccination”, 0 dpv), before challenge (“pre-challenge”, 69 dpv), and 24 hours post-challenge (70 dpv). All animals from the control group showed anti-*C. chauvoei* antibody levels lower than the *cut-off* value throughout the whole study. Meanwhile, all vaccinated animals showed an increase in specific antibody levels after vaccination, albeit significant interindividual variation among them (**Figure 1A**). The antibody level means were not affected by the challenge, except for two individuals (#V6 and #V8), which experienced a drop in antibody levels after challenge. In particular, bovine #V6 showed a titer even lower than the *cut-off* value (**Figure 1A**). Given the high spread of individual responses within the vaccinated cohort, we decided to evaluate whether this variation permitted stratification into high and low responders, we conducted two multimodal statistical analyses. Pre-challenge antibody levels in vaccinated cattle revealed significant bimodality (Silverman’s multimodality test: p = 0.021; Hartigan’s dip test: p = 0.048), indicative of two distinct subpopulations (peak titers: 61.58 and 92.15; **Supplementary Figure 2**). Cluster analysis corroborated this finding, partitioning the cohort into two subgroups (**Supplementary Figure 3**), with significantly different means (Mann-Whitney test, p = 0.0286), that were classified as high responders (n = 4) and low responders (n = 4) based on pre-challenge antibody levels (**Figure 1B**). After challenge, stratification into high and low responders was not observed (**Figure 1C**).

### 3.3 Vaccination elicits antigen-specific cytokine gene expression profile, which is modified after the challenge

**Figure 2:**
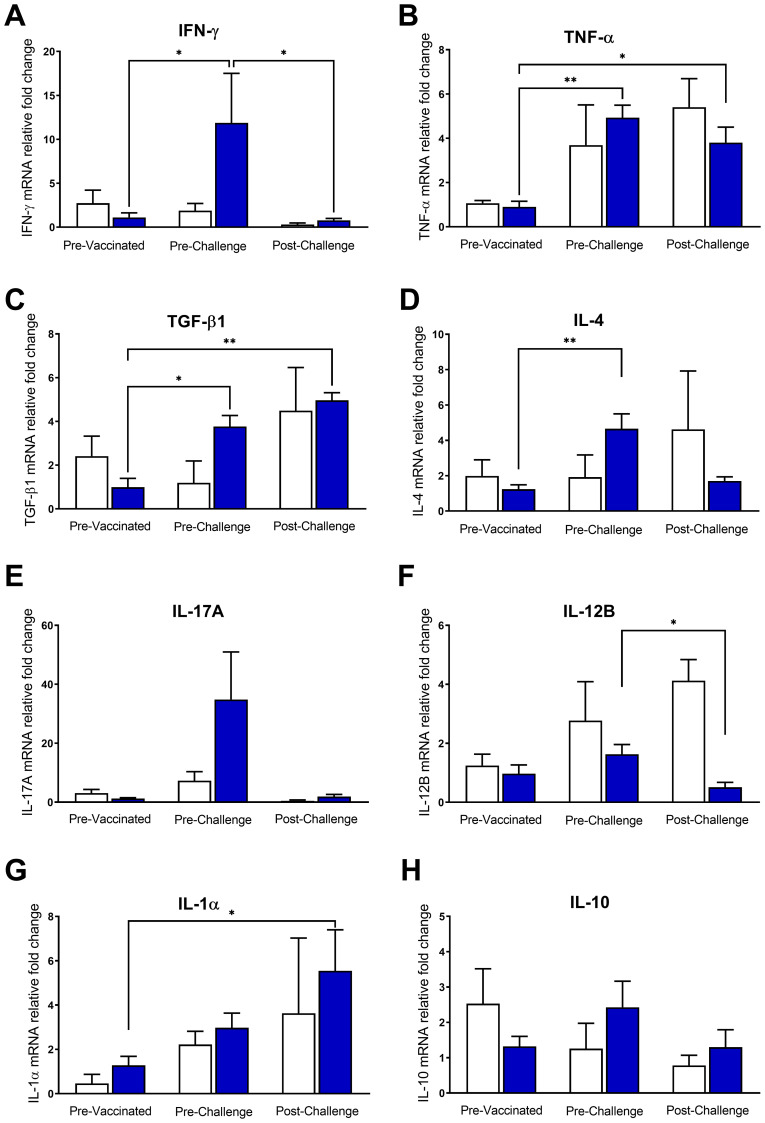
Cytokine gene expression profile in vaccinated and control cattle. Relative mRNA levels of cytokines **(A)** IFN-γ, **(B)** TNF-α, **(C)** TGF-β1, **(D)** IL-4, **(E)** IL-17, **(F)** IL-12B, **(G)** IL-1α and **(H)** IL-10 were quantified at different time points (pre-vaccination, pre- and post-challenge) in whole blood of animals from the vaccinated and control groups stimulated with *C*. *chauvoei* antigen. For each cytokine, relative mRNA levels were expressed relative to the average of the unstimulated condition (PBS) of all cattle (vaccinated and unvaccinated) at 0 dpv (calibration condition). Results show mean ± SEM for vaccinated (blue bar) and control (white bar) groups. Temporal comparisons within each group were assessed using nonparametric paired tests Friedman test, followed by Dunn’s *post hoc* test. Intergroup comparisons (vaccinated vs. unvaccinated) were performed using the Mann-Whitney test. (*p < 0.05; **p < 0.01).

We assessed cytokine gene expression changes in bovine blood samples stimulated *in vitro* with *C. chauvoei* antigen. It is worth to mention that control animals did not experience any change in cytokine response, nor even after challenge. On the contrary, vaccinated animals upregulated the expression levels of IFN-γ, TGF-β1, and IL-4 mRNAs upon antigenic stimulation (**Figures 2A, C, D**). TNF-α also showed an increase after vaccination, but the unvaccinated group showed a similar trend (**Figure 2B**). Although IL-17A mRNA expression levels displayed a tendency toward upregulating transcriptional levels in response to vaccination, this variation lacked statistical significance (**Figure 2E**). Instead, the expression levels of IL-12B, IL-1α, and IL-10 were clearly not modified by vaccination (**Figures 2F–H**).

Conversely, after the challenge, a significant decrease in IFN-γ and IL-12B mRNA expression levels was observed (**Figures 2A, F**). The expression pattern of IL-4 and IL-17A also showed a trend toward downregulation after the challenge (**Figures 2D, E**). However, the expression levels of TNF-α, TGF-β1, and IL-1α mRNAs remained significantly upregulated in vaccinated cattle after the challenge (**Figures 2B, C, G**). IL-10 mRNA expression levels remained unchanged throughout the experiment (**Figure 2H**).

Strikingly, despite the marked temporal differences of some cytokine levels between the vaccinated and unvaccinated animal, there were not statistically significant differences between both groups (**Figure 2**).

### 3.4 Correlation between immunological parameters and clinical outcomes

We then performed correlation analysis to evaluate whether protection was associated with the immunological parameters analyzed in this work. For this, pre- and post-challenge levels of cytokine expression and antibody titers were compared in protected vaccinated *vs* non-protected unvaccinated cattle after the *C. chauvoei* challenge. The analysis was carried out considering individual variations, evaluated as the ratio of the expression levels of cytokines, or the antibody levels, in the Pre-Challenge and Post-Challenge time points, normalized to day 0 (69 dpv/0 dpv and 70 dpv/0 dpv, respectively).

**Figure 3:**
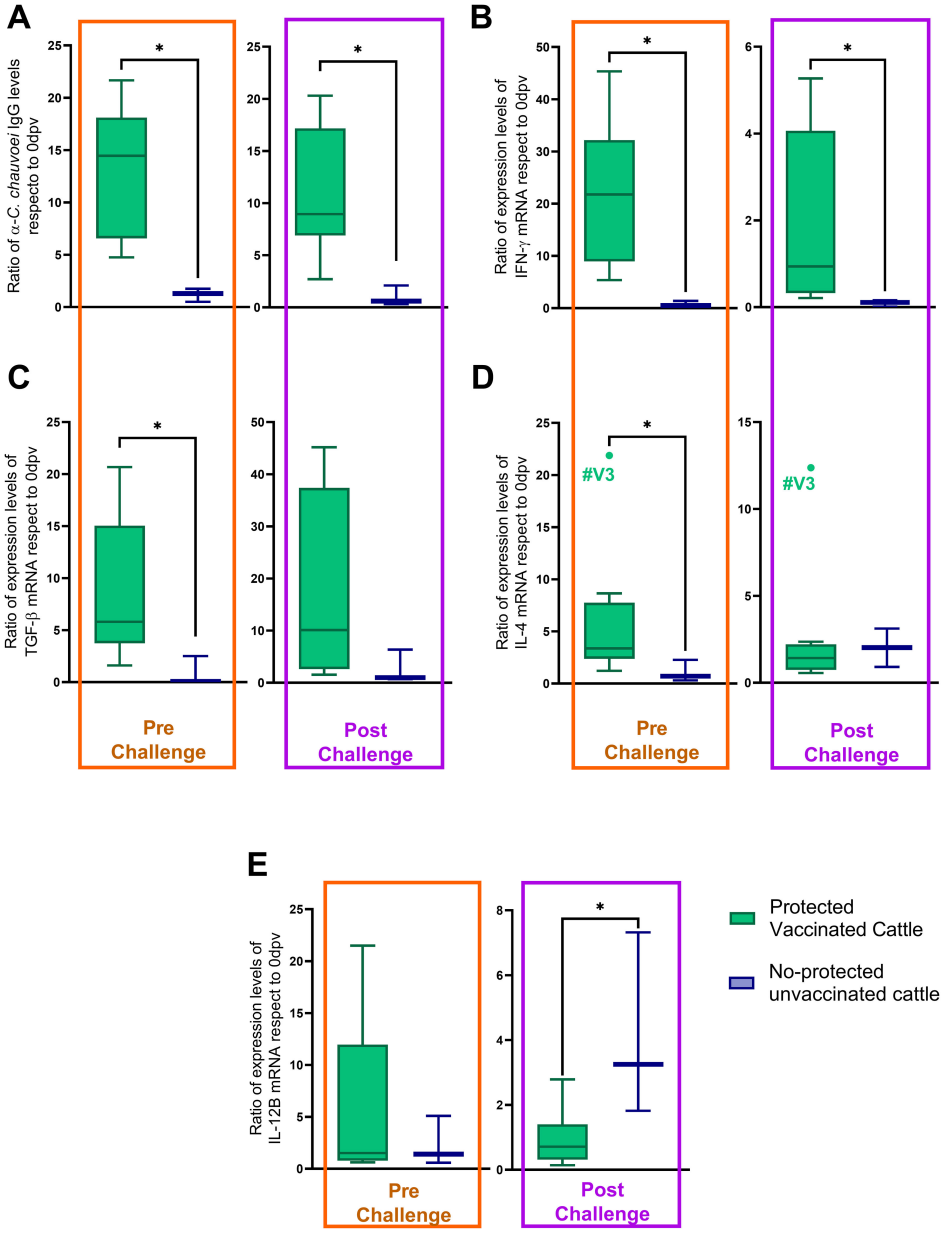
Pre- and post-challenge ratios of immunological parameters in protected vaccinated and no-protected unvaccinated cattle. All ratios (antibody levels or cytokine mRNA expression levels) were normalized to baseline values (0 dpv) for individual animals. **(A)** α-C. chauvoei IgG levels, **(B)** IFN-γ, **(C)** TGF-β1, and **(D)** IL-4, and **(E)** IL-12B mRNA expression levels. Statistical analysis was performed using the Mann-Whitney test (*p < 0.05).

The results showed that the IgG antibody response, as well as IFN-γ, TGF-β1, IL-4, and IL-12B expression levels, had a differential performance between protected and non-protected animals, in at least one time point (**Figure 3**). An increase (higher ratios) in antibody titers and IFN-γ expression levels, both pre- and post-challenge, were associated with protection (**Figures 3A, B**). Equally, an increase in TGF-β1 and IL-4 expression levels before the challenge were also associated with protection (**Figures 3C, D**). In contrast, the results showed that non-protection was associated with an increase in IL-12B expression levels after the challenge (**Figure 3E**). No changes were observed in TNF-α ratios between vaccinated protected and unprotected and unvaccinated subjects.

**Figure 4:**
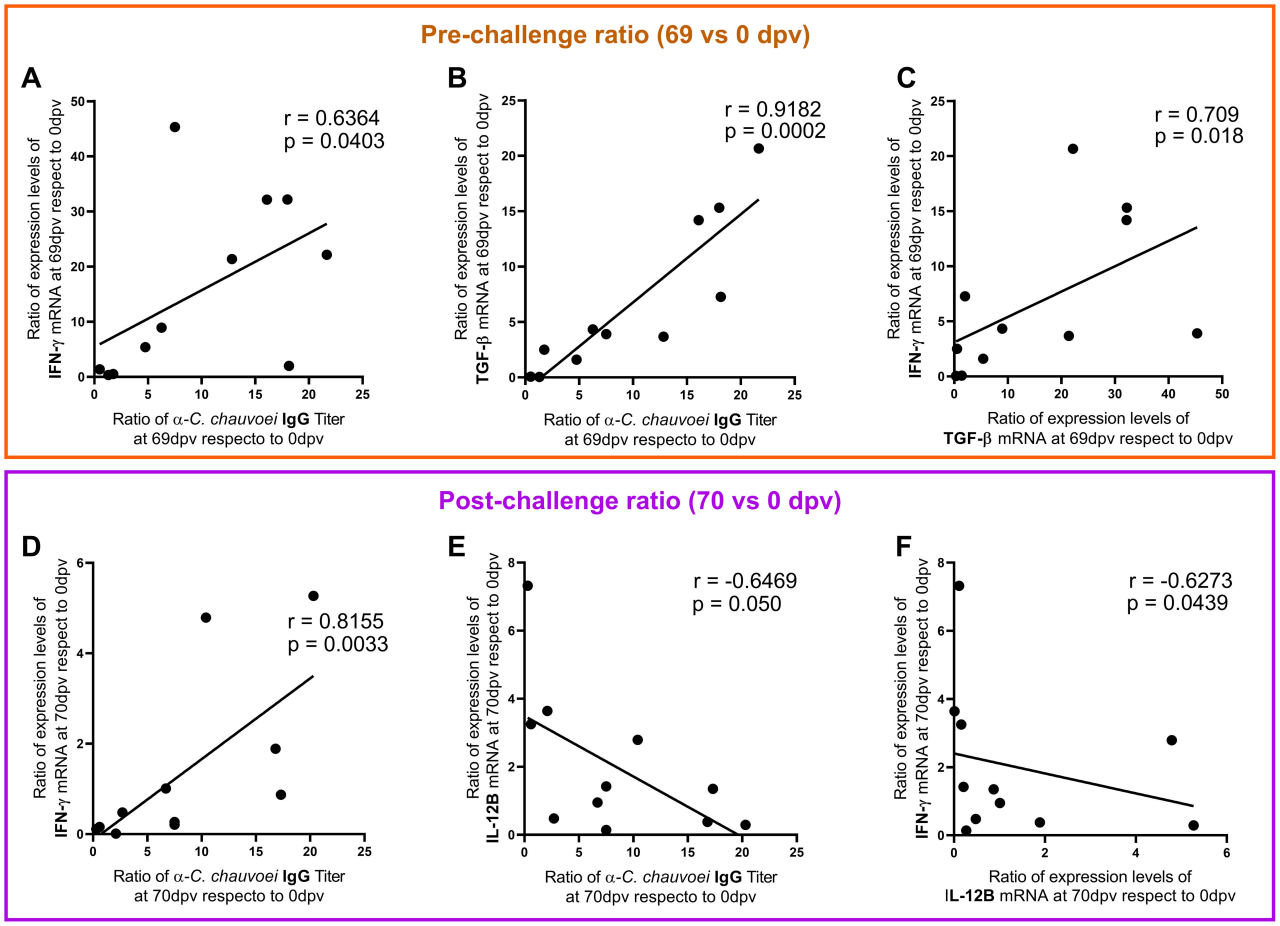
Correlation among immunological parameters at pre- and post-challenge. The graphs depict the correlations among the antibody levels ratio and cytokine gene expression ratio, at **(A–C)** pre-challenge (69 dpv/0 dpv) and at **(D–F)** post-challenge (70 dpv/0 dpv). Spearman linear correlation coefficient (r) and the significance of the correlation (p-value) are shown in each graph. A p-value < 0.05 was considered to indicate statistical significance in all cases.

Additionally, we studied the correlation between these five parameters. The analysis showed a strong positive association between IFN-γ, TGF-β1, and the IgG antibody response before the challenge (**Figures 4A–C**). After the challenge, an association between IFN-γ, IL-12B, and the IgG antibody response was observed, but no correlation involving TGF-β1 was found (data not shown). While the correlation between IFN-γ and the antibody response remained positive (**Figure 4D**), IL-12B negatively correlated with both the antibody response and IFN-γ (**Figures 4E, F**).

### 3.5 Proliferation capacity of vaccinated animals is inhibited after the challenge

Finally, we investigated the lymphocyte (CD4^+^ T, CD8^+^ T, and B cells) proliferation capacity in response to Con A mitogen, as a measure of overall immune cell viability and responsiveness. In samples obtained before the challenge, all three lymphocyte cell populations studied proliferated upon Con A stimulation irrespective of animal vaccination status. Strikingly after the challenge, the proliferation of CD4^+^ and CD8^+^ T cells was decreased in vaccinated cattle (**Supplementary Figures 4A, B**). No changes were observed in B cell proliferation before and after the challenge (**Supplementary Figure 4C**). Furthermore, no differences were observed in the percentages of CD4^+^, CD8^+^ T, or CD22^+^ (B cells) lymphocyte populations pre- and post-challenge (**Supplementary Figures 4D, E**).

## 4 Discussion

Clostridial vaccination has been extensively used to prevent clostridiosis in cattle, including blackleg and gas gangrene caused by *C. chauvoei*. However, until now there is scarce knowledge regarding *C. chauvoei* virulence factors and its mechanism of action, particularly regarding the immune response underlying the protection conferred by *C. chauvoei* current vaccines in cattle (5). This lack of information has hindered the development of better and rationally designed vaccines against this pathogen. In this work, we assessed the IgG antibody response, and for the first time, the cellular immune response elicited in cattle after *C. chauvoei* vaccination and challenge.

As expected, all vaccinated animals survived the *C. chauvoei* lethal challenge and did not show severe symptoms, while all unvaccinated cows showed severe symptoms and died after challenge (**Table 2**).

All vaccinated cattle elicited high levels of anti-*C. chauvoei* IgG antibodies (**Figure 1A**), although with significant interindividual variability (**Figure 1B**), consistent with previous findings by other authors (13, 32–34). Before the challenge, vaccinated animals could be stratified into high and low IgG antibody responders. Kerry and Craig and we have previously described a similar behavior in vaccinated sheep (11, 32). To the best of our knowledge, this is the first report of the heterogeneity in the humoral immune response against *C. chauvoei* in cattle. Many factors may influence the IgG immune response elicited by the host upon vaccination, such as genetic factors, age, sex, nutritional status, concomitant diseases, environmental factors, and stress among others (35, 36). After the challenge, the IgG immune response remained unchanged for all vaccinated animals (**Figure 1A**), except for two bovines, which showed a marked decrease in their IgG levels (**Figure 1C**). Interestingly, for #V6 that was classified as a low responder prior to the challenge, its antibody titer declined sharply 24 hours post-challenge, reaching levels below the established *cut-off* value. This decrease could be explained by the fact that those circulating antibodies could be interacting with *C. chauvoei* antigens, and thus, would be sequestered and not detected in sera. However, no link with symptomatology was found in this case.

On previous work we reported that IgG titers induced by polyclostridial vaccines in sheep and cattle are not long-lasting; becoming undetectable within 6 months after vaccination (11, 12). This finding suggests that, while the humoral immune response plays a fundamental role in protection against *Clostridium* spp., the cellular immune response would also be essential to achieve and maintain this protection. Therefore, we also assessed the cellular immune response elicited against vaccination and lethal challenge with *C. chauvoei* in cattle. The temporal evolution of some cytokine’s levels showed clear differences between vaccinated and unvaccinated cattle; however, no significant differences were detected for the cytokines evaluated between the vaccinated and unvaccinated groups or at any of the time points analyzed (pre-vaccination, pre-challenge, and post- challenge). This was a unexpected result, most likely due to the small size of the unvaccinated group. We found that the polyclostridial vaccine induced a cellular immune response against *C. chauvoei*, which includes proinflammatory and regulatory responses, with high levels of IFN-γ, TNF-α, TGF-β1, and IL-4 mRNA expression (**Figure 2**). The high levels of IFN-γ expression in vaccinated cattle (**Figure 2A**) are in agreement with other reports showing a specific IFN-γ response to polyclostridial vaccination. Sheep vaccinated with a polyclostridial vaccine showed increased IFN-γ mRNA expression in PBMCs stimulated with *C. chauvoei* antigen (20). Cattle vaccinated against *C. difficile* (35), and mice vaccinated against *C. perfringens* (37), both exhibit increased IFN-γ expression in response to vaccination. Likewise, an increase in IFN-γ has been reported in other clostridial infections, such as *C. tetani* in humans (25), *C. difficile* infection in humans (21, 22), *C. perfringens* in pigs (23) and chickens (24). The relevance of IFN-γ in the response against *C. chauvoei* could be associated with its ability to induce inflammatory response by activating macrophages (2, 16, 18, 19, 38) and recruiting neutrophils (39–42), thereby enhancing bacterial elimination. Regarding TNF-α, it is known to be the main inducer of inflammation and is produced by innate immune cells and by effector T cells, helping in the recruitment of lymphocytes, macrophages, and neutrophils (43). Previously, it was reported that the vegetative form of *C. chauvoei* induces the expression of TNF-α in murine macrophages (19). Others have reported that TNF-α is also induced by other *Clostridia* such as *C. perfringens*, *C. histolyticum*, *C. clostridioforme*, *C. leptum*, *C. porosphaeroides* (44) and *C. sordelli* (45). Although our study showed an increase in TNF-α levels following vaccination, no definitive conclusions can be drawn regarding this cytokine, as the unvaccinated control group exhibited a similar trend (**Figure 2B**), and the limited available data come from models different from the one used in our study. In addition, we found an increase in TGF-β1 expression in vaccinated cattle. TGF-β1 is a critical anti-inflammatory immunomodulator that regulates and limits inflammation by suppressing the differentiation of T lymphocytes (Th1, Th2, and cytotoxic), and NK cells and inhibiting B cell proliferation (46). In this context, TGF-β1 would play an important role in maintaining the balance between the vaccine-induced inflammatory response and the detrimental effects of exacerbated inflammation. On the other hand, when TGF-β1 is associated with IL-6 or IL-1β, it has been reported to promote a Th17 differentiation (43, 46). Our results showed a trend towards an increase in IL-17 expression levels in response to polyclostridial vaccination (**Figure 2E**). In this regard, it would be interesting to evaluate the expression of IL-6 or IL-1β cytokines to confirm whether TGF-β1 could be playing an anti- or pro-inflammatory role in response to *C. chauvoei*. Surprisingly, the proinflammatory response against *C. chauvoei* induced by vaccination was accompanied by an increase in IL-4 expression (**Figure 2D**). The upregulation of this cytokine, typically associated with Th2 responses, could be explained because in cattle there are antigen-specific double-positive CD4^+^ T cells (IFN-γ^+^ IL-4^+^), called Th0, capable of coproducing distinctive cytokines associated with Th1 and Th2 response. In cattle, Th0 cells act as early-stage immune cells and serves as a critical precursor to effector T helper subsets (Th1, Th2, Th17, Treg). Their role is well studied in humans and mice but not in cattle. Their plasticity may influence outcomes in infections or vaccinations, particularly in balancing pro-inflammatory and regulatory responses. However, mechanistic insights into bovine Th0 regulation, including transcriptional regulation and epigenetic modifiers, are scarce. Further research is needed to clarify their contributions to protective immunity in cattle, with implications for vaccine design and therapeutic strategies targeting immune polarization (47).

A potential source of cytokines is γδ T cells, which constitute 30-60% of PBMCs in young cattle and exhibit an early response similar to innate immune cells. In ruminants, γδ T cells can produce IFN-γ, IL-17, IL-10, and TGF-β, as well as exhibit cytotoxic activity and memory responses (reviewed in (48)). These cells have been associated with infections or vaccination against various viruses, bacteria, and protozoa (reviewed in (49)). In this work, we were unable to characterize this cell type and we consider that it will be interesting to address this cell population in future studies.

When then assessed whether vaccination-induced protection (pre-challenge time) is associated with the immunological parameters analyzed in this study (antibody and cytokine levels), the analysis demonstrated that protected vaccinated cattle exhibited significantly elevated IgG anti-*C. chauvoei* and IFN-γ, TGF-β1, and IL-4 mRNA levels in response to *C. chauvoei* antigens than non-protected unvaccinated animals (**Figure 3**). Notably, a positive correlation was identified between IFN-γ and TGF-β1 expression levels and IgG antibody titers (**Figure 4A**). These results suggest a link between elevated IgG/IFN-γ/TGF-β1 and protection, though further studies are needed to establish causality. These findings support our hypothesis that vaccine-induced protection involves coordinated humoral (antibody-driven) and cellular (cytokine-mediated) immune responses. While antibody titers remain the established primary correlate of vaccine efficacy, our results indicate that cellular immune mediators, such as IFN-γ and TGF-β1, may also function as potential correlates of protection against *C. chauvoei*. This highlights the importance of evaluating both humoral and cellular immunity in comprehensive assessments of vaccine-induced immune responses.

It is important to note that, although the observed correlations underscore the potential relevance of certain cytokines, none of the cytokines analyzed allowed for the classification of animals as high or low responders (data not shown), not could they be associated with the groups defined by humoral immune response. Moreover, we did not identify any consistent cytokine expression pattern that could explain the decrease in antibody titers observed in animals #V6 and #V8 (data not shown). Although both animals retained the capacity to mount a cellular immune response following challenge, they exhibited variable expression levels across all cytokines assessed. We hypothesize that the reduction in humoral response may be linked—either directly or indirectly—to a broader set of immunological parameters not included in this study. To uncover potential associations, future research should involve a more comprehensive analysis of immune markers or employ high-throughput approaches such as RNA sequencing.

Following a lethal challenge with a suspension of *C. chauvoei* spores, a different immune response profile was observed. *C. chauvoei* spores can remain dormant until anaerobic conditions favorable for their germination are present at the muscle level. Traditionally, pathogenic clostridia were considered exclusively extracellular pathogens. However, studies have demonstrated that *C. chauvoei* can survive within bovine PBMC-derived macrophages, similar to findings with *C. difficile* and *C. perfringens*, which can persist within murine macrophages for up to 72 hours post-infection (17, 18, 38). Pires and collaborators reported that macrophages infected with vegetative forms of *C. chauvoei* induced a pro-inflammatory gene expression profile characterized by IL-12 and IL-23, cytokines that drive Th1 and Th17 immune responses, respectively. In contrast, macrophages infected with *C. chauvoei* spores exhibit an anti-inflammatory profile dominated by IL-10 and TGF-β expression (16). It has been proposed that macrophages may fail to recognize spores due to their low metabolic activity, allowing spores to utilize these cells for survival and dissemination without eliciting an inflammatory response (2, 16–18, 38). Our results indicate that non-protected unvaccinated cattle experience greater ratio in IL-12B expression levels and lower ratio IgG titers and IFN-γ expression levels upon challenge, compared to protected vaccinated cattle (**Figure 3**). Unlike the approach used by Pires et al. (16), our experimental design does not allow us to determine which cell population is responsible for producing these cytokines. However, our findings suggest that IL-12B expression is associated with a worse post-challenge clinical prognosis. In this context, we hypothesize that, 24 hours after challenge, the spores would have already germinated, and as a result, macrophages infected with the vegetative form of *C. chauvoei* would be expressing IL-12B. In this study, we quantified IL-12B expression alone, which is common to both IL-12 and IL-23. Further investigation into IL-12A and IL-23 expression would be valuable to confirm whether both cytokines are induced during infection. All in all, our findings suggest that IL-12B expression is associated with a worse clinical prognosis following exposure to *C. chauvoei* spores, while vaccine-induced protection relies on *C. chauvoei*-specific IgG and IFN-γ, even though their levels decrease post-challenge. Strikingly, the association between IgG and IFN-γ was maintained after the challenge, whereas a negative correlation between these parameters and IL-12B emerged (**Figure 4B**). The post-challenge results suggest a relationship between IgG/IFN-γ/IL-12B and protection, although further studies are needed to establish causality. Interestingly, different pre- and post-challenge correlations are established, with an anti- or pro-inflammatory role for TGF-β1 pre-challenge, while post-challenge IL-12B acquires a pro-inflammatory role associated with *C. chauvoei* infection (**Figure 4**).

In addition, we assessed the response to Con A stimulation, as measure of overall proliferative capacity of αβ T and B cells after vaccination and *C. chauvoei* challenge. Vaccination did not alter the non-specific proliferative capacity of these cells (**Supplementary Figure 4A**), however, protected animals showed a decreased proliferative capacity of CD4^+^ and CD8^+^ αβ T lymphocytes 24 hours post-challenge, concomitant with dysregulated mRNA expression of cytokines—predominantly IFN-γ (**Figure 2A**). Although this was an unexpected result, a similar phenomenon has been reported during moderate sepsis events, where memory CD8^+^ T cell populations, typically stable under homeostatic conditions, undergo numerical depletion and functional impairment (reviewed in (50)). It could be that the observed reduction in antigen-specific IFN-γ production and proliferative capacity may reflect analogous immunosuppressive mechanisms. However, mitogen-based assays such as those using Con A do not specifically assess antigen-experienced memory T cell responses. Therefore, to investigate this hypothesis, further studies employing antigen-specific approaches, such as peptide–MHC tetramer staining or cytokine profiling following antigen restimulation, are required to clarify the functional status of memory T cells and confirm a potential association.

In an endotoxemia model in humans, it has been demonstrated that as early as 3 hours after LPS inoculation, whole populations of CD4^+^ and CD8^+^ T cells decline, and the pro-inflammatory T-helper cells transiently lose their capacity to produce IL-2, TNF-α, as well as IFN-γ, whereas IL-10 production was unaffected. This impairment was restored 24 hours post-LPS inoculation (51). Although in this work we assessed the expressions of these cytokines only 24 hours post-challenge (and not as early as 3 hours post-challenge), the *C. chauvoei* challenge is not as an acute model as it is the LPS inoculation, and thus it can be hypothesized that the same phenomenon is occurring but not as fast as in the endotoxemia model, as we have shown the same behavior with the same cytokines and in the same T cell subsets.

While the mechanisms underlying these events remain elusive, it can be hypothesized that it occurs as a protective mechanism to prevent the extensive tissue damage and the resultant cytokine storm that occurs during a severe sepsis event, that may lead to multiorganic failure and death. Since this mechanism was reported in other mammals, such as mice and humans, it makes sense that also in bovines a decrease in the elicited cellular immune response promotes homeostasis, and thus, plays a key role in overall protection. While this was reported in certain effector subpopulations of T CD4^+^ and CD8^+^ cells, in this work we were not able to further characterize the involved populations, but certainly, it is an interesting approach to address shortly. Additionally, investigating changes in γδ T cell populations could provide valuable insights into their potential role in the immune response elicited by these vaccines.

In summary, this is the first report on the induction of a protective cellular immune response induced by a polyclostridial vaccine against *C. chauvoei* in cattle. Protection is associated with humoral and cellular immune responses, which include proinflammatory and regulatory components, since IFN-γ and TGF-β1 were detected. IL-12B expression is associated with a worse clinical prognosis when challenged with *C. chauvoei* spores. All in all, these results provide considerable knowledge to further understand the immune response underlying protection against a deadly pathogen such as *C. chauvoei*, for which current knowledge is scant. Our results show that IFN-γ expression may be considered as a biomarker of clostridial vaccination together with antibody titer. IFN-γ as a biomarker would provide a more appropriate tool to further study cellular responses and long-term memory. In this sense, our results enable a better comprehension of the immune response conferred by the current clostridial vaccines against *C. chauvoei* and shed light to advance toward the development of better and rationally designed *C. chauvoei* vaccines.

## Data Availability

The original contributions presented in the study are included in the article/**Supplementary Material**. Further inquiries can be directed to the corresponding author.
