# Supplementary material for: Humoral and cellular immune responses in cattle upon Clostridium chauvoei vaccination and challenge
Source: Front Immunol. 2025 May 20;16:1584168. doi: 10.3389/fimmu.2025.1584168 (PMC12130040; doi:10.3389/fimmu.2025.1584168)

Supplementary Figure 1. Gating strategy to analyze the proliferative capacity of PBMC from vaccinated and unvaccinated cattle before and after challenge. First, the cells were identified and gated based on the FSC-A *vs.* SSC-A parameters (P1), as observed in the corresponding dot plot. Subsequently, doublets were excluded by examining the FSC-H *vs.* FSC-A dot plot (P2). Next, the CD4⁺ T lymphocytes (CD4-PE), CD8⁺ T lymphocytes (CD8-Alexa 647) and B lymphocytes (CD22-biot_PECy7) positive cells were gated within P2, as demonstrated in FSC-A *vs.* CD8 APC (P12) dot plot, FSC-A *vs.* CD4 PE-A (P11) dot plot, and FSC-A *vs.* CD22 PE-Cy7-A (P34) dot plot. Finally, CFSE staining within each cell population was obtained and the different generation of cells were gated (e.g., cell generation gates for CD8+ were gated P3 to P11). The data in the figure are shown as an example and representative of all samples analyzed. Analysis was performed using BD FACSDiva Version 6.1.3 software.

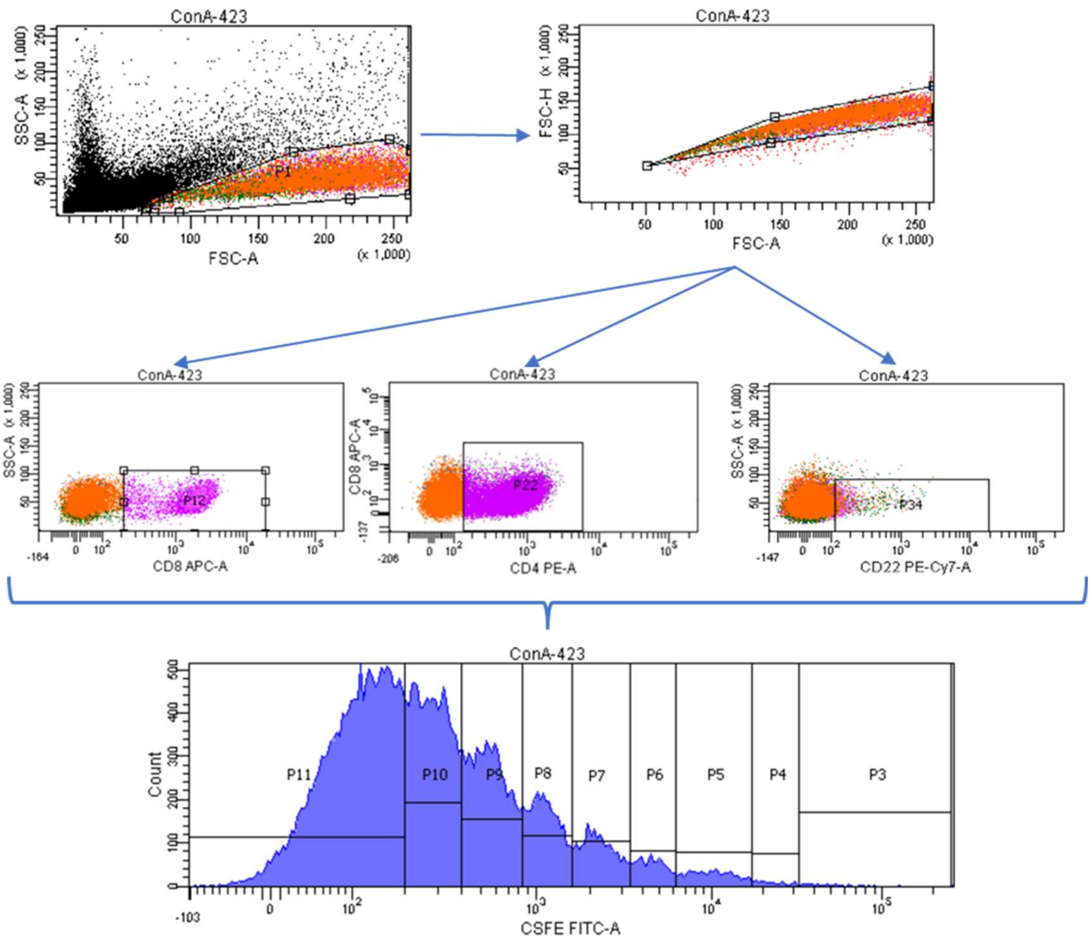

Supplementary Figure 2. Pre-challenge antibody titers in vaccinated cattle revealed significant bimodality. The figure displays a probability density curve generated using a Gaussian Kernel function based on the pre-challenge antibody titers. Two local maxima (modes) were identified at approximately 61.58 and 92.15, as indicated by red points and dashed vertical lines. Pre-challenge antibody titers in vaccinated cattle revealed significant bimodality (Silverman's multimodality test: p = 0.021; Hartigan's dip test: p = 0.048). The analysis was conducted using R statistical software.

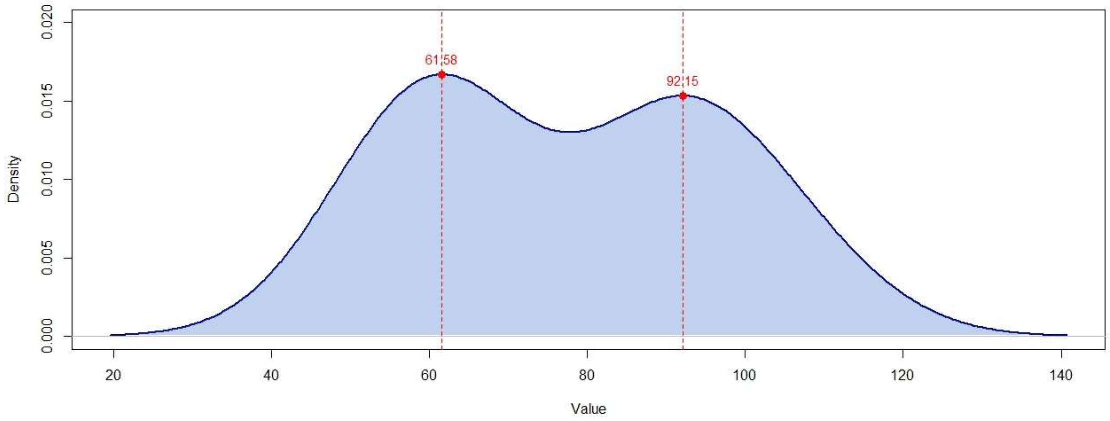

Supplementary Figure 3. Pre-challenge vaccinated group is divided into two subgroups. K-means clustering (k = 2) of Z-score-normalized antibody levels segregated the pre-challenge vaccinated group into two distinct clusters: Cluster 1 (blue) and Cluster 2 (orange). Each dot represents an individual pre-challenge vaccinated bovine, colored according to its assigned cluster. The dotted lines indicate the centroids of Clusters 1 and 2, respectively. The analysis was conducted using R statistical software

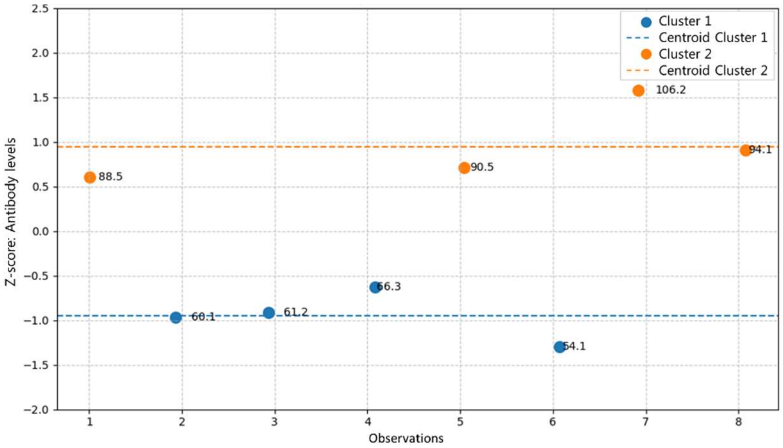

Supplementary Figure 4. Pre- and post-challenge proliferation of CD4⁺ T, CD8⁺ T and B lymphocytes in vaccinated and control cattle. CFSE-labeled PBMC from vaccinated (*n* = 5) and control cattle (*n* = 3) were *in vitro* stimulated with ConA or left unstimulated. Proliferation was analyzed by flow cytometry at pre- and post-challenge time points. Lymphocyte populations were identified using CD4-PE ($CD4^+$ T cells), CD8-Alexa 647 ($CD8^+$ T cells), and CD22-biot/Strp-PECy7 (B cells). **(A–C)** Violin-boxplots representing the proliferation index (PI) of **(A)** $CD4^+$ T cells, **(B)** $CD8^+$ T cells, and **(C)** B cells in vaccinated cattle (ConA: dark pink; unstimulated: light pink) and control cattle (ConA: dark green; unstimulated: light green). Statistical analysis was performed using the Wilcoxon test (**$p < 0.01$). **(D)** Percentage of total lymphocytes in control (Orange) and vaccinated (blue) cattle at pre- (solid bar) and post-challenge (striped bar) times points. Data are presented as mean ± SEM. Statistical analysis was performed using the Wilcoxon test. **(E)** Percentages of $CD4^+$ T (light blue bar), $CD8^+$ T (grey bar), and B (green bar) cells within the total lymphocyte population (100%). Statistical analysis was performed using the Wilcoxon test.

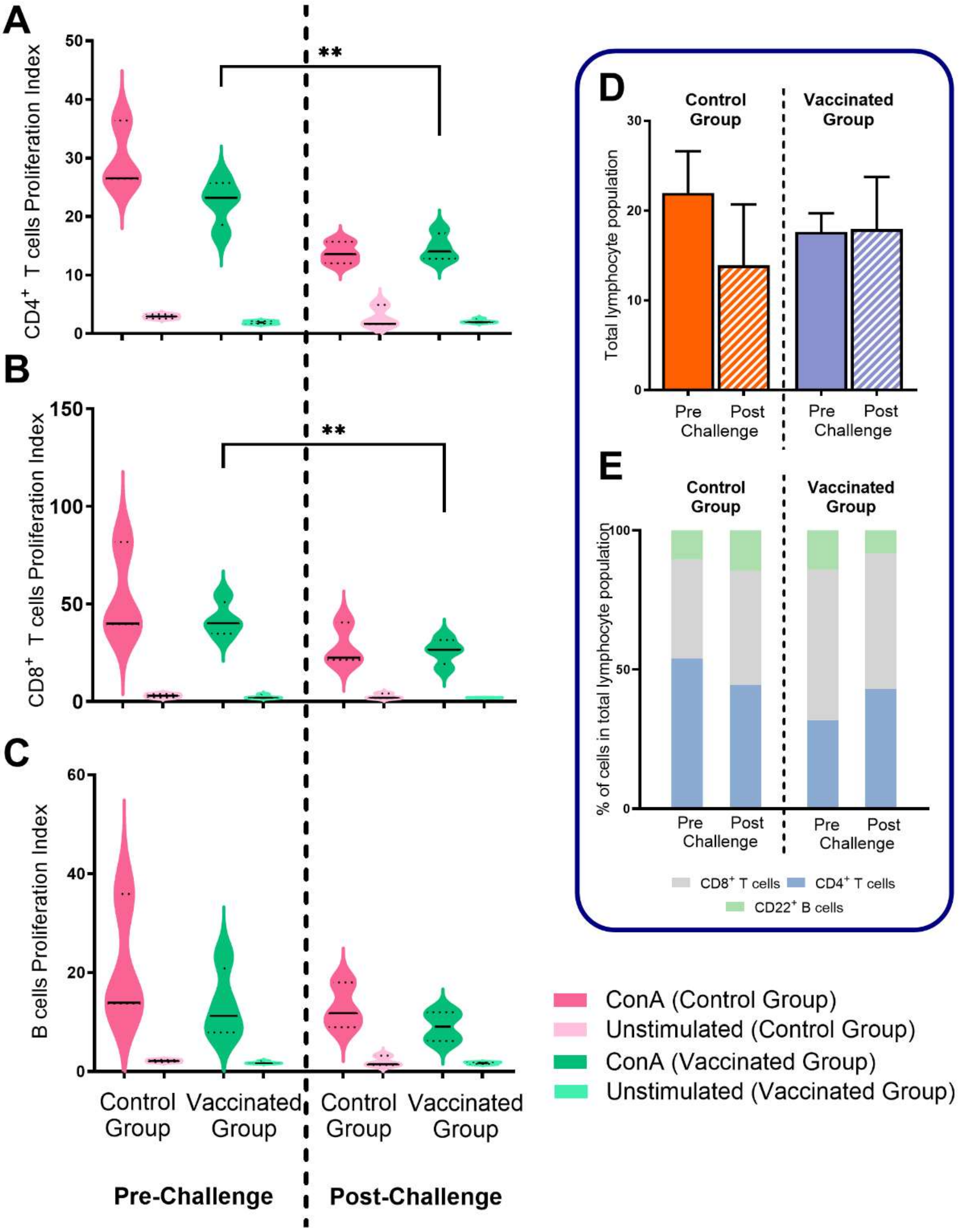

Supplement: Supplementary file 1 [file DataSheet1.pdf]
